# On the Importance of Countergradients for the Development of Retinotopy: Insights from a Generalised Gierer Model

**DOI:** 10.1371/journal.pone.0067096

**Published:** 2013-06-27

**Authors:** David C. Sterratt

**Affiliations:** Institute for Adaptive and Neural Computation, School of Informatics, University of Edinburgh, Edinburgh, Scotland, United Kingdom; Indiana University, United States of America

## Abstract

During the development of the topographic map from vertebrate retina to superior colliculus (SC), EphA receptors are expressed in a gradient along the nasotemporal retinal axis. Their ligands, ephrin-As, are expressed in a gradient along the rostrocaudal axis of the SC. Countergradients of ephrin-As in the retina and EphAs in the SC are also expressed. Disruption of any of these gradients leads to mapping errors. Gierer's (1981) model, which uses well-matched pairs of gradients and countergradients to establish the mapping, can account for the formation of wild type maps, but not the double maps found in EphA knock-in experiments. I show that these maps can be explained by models, such as Gierer's (1983), which have gradients and no countergradients, together with a powerful compensatory mechanism that helps to distribute connections evenly over the target region. However, this type of model cannot explain mapping errors found when the countergradients are knocked out partially. I examine the relative importance of countergradients as against compensatory mechanisms by generalising Gierer's (1983) model so that the strength of compensation is adjustable. Either matching gradients and countergradients alone or poorly matching gradients and countergradients together with a strong compensatory mechanism are sufficient to establish an ordered mapping. With a weaker compensatory mechanism, gradients without countergradients lead to a poorer map, but the addition of countergradients improves the mapping. This model produces the double maps in simulated EphA knock-in experiments and a map consistent with the Math5 knock-out phenotype. Simulations of a set of phenotypes from the literature substantiate the finding that countergradients and compensation can be traded off against each other to give similar maps. I conclude that a successful model of retinotopy should contain countergradients and some form of compensation mechanism, but not in the strong form put forward by Gierer.

## Introduction

During late prenatal and early postnatal neural development in vertebrates the axons from retinal ganglion cells (RGCs) grow and are pruned so as to form a topographic mapping from the retina to its target regions. To explain how regenerating fibres in goldfish innervate the appropriate part of tectum [1], Sperry proposed that the establishment of the map depends on retinal and target cells expressing varying levels of biochemical labels that allow growth cones to identify their correct targets by finding cells with a matching or complementary label [2]. Broadly consistent with this *chemoaffinity hypothesis*, during the period in which the map is formed, EphA and EphB receptors are expressed in gradients along orthogonal axes of the retina and their ligands, ephrin-As and ephrin-Bs, are expressed along orthogonal axes of the superior colliculus (SC) or optic tectum, and Eph-ephrin signalling has been shown to have a role in guidance [3].

Much recent work has focused on one dimension of the mapping, from the retinal nasotemporal axis to the rostrocaudal axis of the SC. In mouse and chick, EphA receptors are expressed in a low-to-high gradient along the nasotemporal axis of the retina, and their ligands, ephrin-As, in a low-to-high gradient along the rostrocaudal axis of the SC [3]–[7]. Via *forward* signalling, activation of axonal EphA receptors by ephrin-A expressed in the tectum leads to axon repulsion [5]. There is also expression of ephrin-As along the nasotemporal axis of the retina, but as a *countergradient* to the retinal EphAs, i.e. a gradient in the opposing (high-to-low) direction [7]–[9]. Correspondingly, there is a countergradient (high-to-low) of EphA expressed along the rostrocaudal axis of the SC, in opposition to the ephrin-A gradient. The activation of axonal ephrin-A by EphA in the SC, called *reverse* signalling [10], [11], also inhibits axon growth [12]. Genetic manipulations of EphAs or ephrin-As cause disruptions to the topographic map [12]–[22].

Gierer's models [23]–[25] indicated that matched gradient and countergradient pairs with inhibitory interactions could establish topographic maps. This model and elaborated versions of it [26] are consistent with and provide an explanation for the existence of countergradients [12], [22]. However, the model contains the strong assumption that gradients and countergradients are closely matched, presumably by genetic mechanisms. It has been argued [27] that matched gradients and countergradients alone cannot account for perturbations such as the double maps produced in mice in which extra EphA is expressed in RGCs at random using a knock-in strategy [15], [16]. In contrast, these maps are predicted by models containing gradients with fibre-target forward signalling and adaptive mechanisms such as competition [28], [29] or marker induction [30] but which do not include countergradients with reverse signalling.

Nevertheless, given that genetic manipulations of countergradients cause mapping errors [12], [22], it is important to understand their role in models of map development. The main aim of this paper is to investigate how countergradients and various forms of compensatory mechanism might interact. For this I use a modified version of Gierer's model of 1983 which contains both countergradients and a compensatory mechanism. This 30-year old model has been chosen because, while it is not as comprehensive as more recent models, its simple formulation allows the relative influence of countergradients and adaptive mechanisms to be assayed. Features absent from Gierer's model but present in others include activity [29], [31]–[34], *cis-* fibre-fibre interactions [35], fasciculation and defasiculation effects [36] and induction of collicular gradients [30]. One other advantage of Gierer's model is that it does not require strong assumptions to be made about the tuning of the size and interaction strength of forward and reverse gradients implicit in a number of models [26], [33], [34], [36]. A secondary aim of this paper, motivated by the recommendation that existing models should be tested against new data [37], is to determine whether the Gierer model can account for the recent data derived from EphA3 knock-in [15], [16] and Math5 knock-out [32] phenotypes.

## Results

### Gradients and Countergradients without Compensation do not Ensure Topographic Map Formation

**Figure 1:**
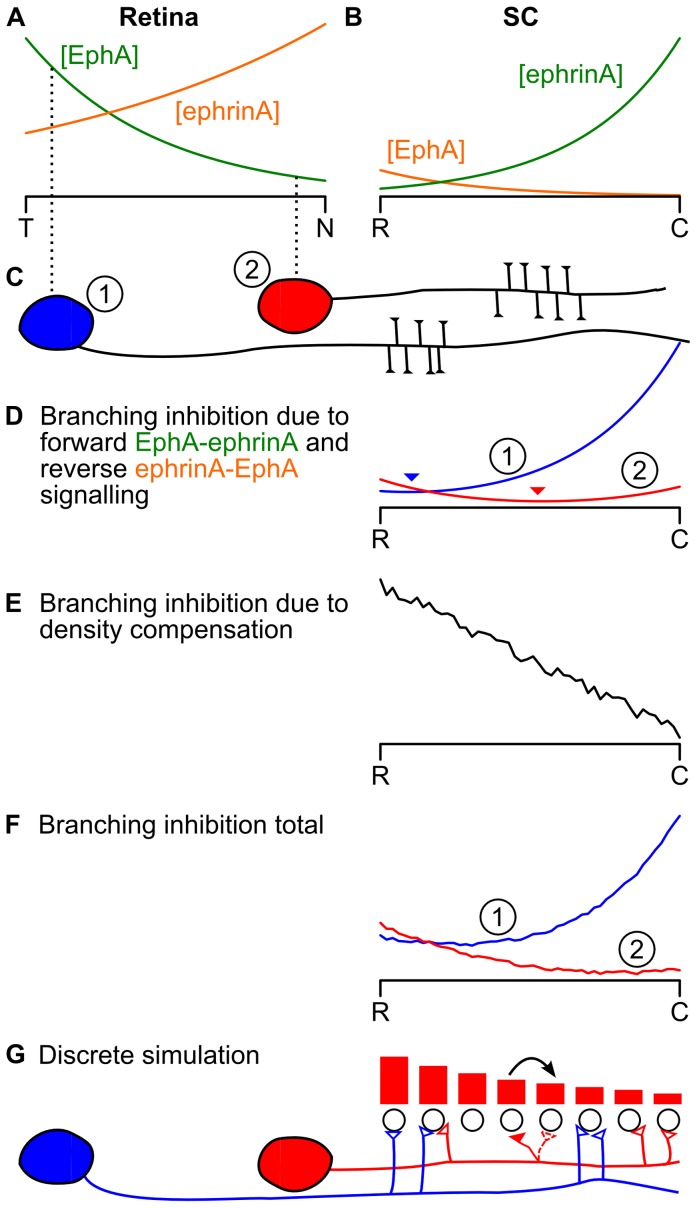
Overview of model. **A, B** Gradients and countergradients of EphA and ephrin-A in the retina and SC. Collectively the retinal EphA and collicular ephrin-A make up the forward system referred to as *gradients* and are indicated in green; the retinal ephrin-A and collicular EphA make up the reverse system referred to as *countergradients* and are indicated in orange. **C** A temporal RGC (1, blue) and a nasal (2, red) RGC and their expected preferred locations of arborisation in the SC shown schematically as side branches tipped with growth cones. **D** The branching inhibition due to the sum of gradient and countergradient signalling experienced by the temporal (blue) and nasal (red) axon along the rostrocaudal axis of the SC. Minima are indicated by arrowheads. **E** The branching inhibition due to density compensation experienced by both axons along the rostrocaudal axis of the SC as the system is approaching its final configuration. **F** The total branching inhibition for each RGC; this is the sum of the corresponding curve in **D** and the curve in **E**. **G** The discrete implementation. A terminal (red filled synapse) is picked at random and moved in the direction of lower total branching inhibition for that axon (indicated by heights of red bars).

The model, depicted in Fig. 1 and detailed in the Models section and Table 1, has a generalised version of the mathematical structure of the 1983 Gierer model [24], but the gradients are interpreted as being EphAs and ephrin-As, which had not been identified in 1983. I make the assumption, justified in the Discussion, that the mapping from the two-dimensional retinal surface to the two-dimensional surface of the superior colliculus (SC) can be simplified by supposing that the mapping from the nasotemporal axis to the rostrocaudal axis occurs independently from the mapping from the dorsoventral axis to the mediolateral axis. I focus on the nasotemporal to rostrocaudal mapping and the associated signalling system of EphAs and ephrin-As because it is better understood than the EphB and ephrin-B signalling associated with the dorsoventral to mediolateral mapping.

**Table 1:** Parametrisation of gradients.

	Retina	SC
**Gradients**	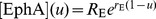	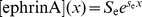
**Countergradients**	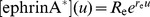	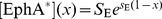

The table shows the expressions for the concentrations [EphA]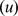 and [ephrinA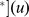 of EphA and ephrin-A at a distance 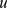 along the nasotemporal axis of the retina and the concentrations [ephrinA]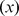 and [EphA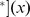 of ephrin-A and EphA at a distance 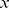 along rostrocaudal axis of the SC. The temporal pole of the retina lies at 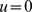 and the nasal pole at 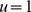. In the SC 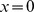 is the rostral pole and 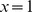 is the caudal pole. The heights of gradients in the retina and SC are denoted by 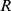 and 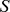 respectively, with a subscript ''E'' or ''e'' to denote whether it is an Eph or ephrin. These subscripts are also applied to the decay or rise constants of retinal and SC gradients, denoted 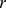 and 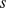 respectively.

Along the nasotemporal axis of the retina (Fig. 1A) there is a low-to-high gradient of EphA and a countergradient of ephrin-A running from high-to-low. Along the rostrocaudal axis of the SC (Fig. 1B) there is a low-to-high gradient of ephrin-A and a high-to-low countergradient of EphA. A temporal retinal ganglion cell (RGC) axon (labelled (1) in Fig. 1C) therefore bears more EphA than ephrin-A, whereas the converse is true of a nasal axon (labelled (2) in Fig. 1C). Via the forward signalling pathway, the EphA on each axon interacts with the ephrin-A on each SC cell to produce a signal that inhibits branching and that is proportional to the product of the densities of EphA on the axon and the ephrin-A on the SC cell. Since the amount of ephrin-A varies throughout the SC, so does the inhibitory signal. The branching inhibition for the reverse signalling pathway is taken to be the product of the densities of ephrin-A on the RGC axon and EphA on the SC cell.

The branching inhibition signals produced by the forward and reverse pathways are summed to produce the net branching inhibition signals for RGC axons (1) and (2) seen in Fig. 1D. The most favourable location for axon (1) to branch is at the rostral end of the SC, where the branching inhibition is lowest. This is the topographically ''correct'' position for this axon. The most favourable location for axon (2) to branch is just over halfway along the rostrocaudal axis; this is not the correct position for this nasal axon, which should connect to the caudal end of the SC.

This shows that in a model in which there are only fibre-target interactions, the gradient and countergradient (or forward and reverse signalling) systems do not necessarily ensure formation of a topographic map. In theory, the parameters of the gradients and countergradients could be matched so that a perfect topographic map is formed (see Models section). However it would seem to be hard to achieve this precise matching biologically and, as can be verified using the simulation method presented later, mismatches can result in the entire colliculus not being covered and/or bunching of connections at one end (data not shown). Furthermore, even if the gradients could be arranged to produce the desired mapping the system would not be robust to surgical manipulations or changes in the gradients, whereas considerable robustness to perturbations have been observed in a variety of species [14]–[16], [38], [39]. A recent model suggests that Eph/ephrin forward and reverse fibre-fibre interactions between RGCs could compensate for mismatched gradients [35]. However, it is not clear if this result depends on a precise matching of the parameters of the fibre-fibre interactions (see Discussion).

### Strong Compensation with Gradients but no Countergradients Produces a Topographic Map

To account for expansion and contraction experiments [38], [39], Gierer [24] proposed adding a mechanism, which he called ''regulation'', to the model described so far. The use of the term ''regulation'' is unfortunate as it has a specific meaning in developmental biology, so for clarity I use the term ''compensation''. The idea entails axonal growth cones inhibiting each other's growth by releasing an inhibitory substance that builds up over time. The greater the density of growth cones in a small region of the SC, the harder it is for growth cones to make connections there. For example, in Fig. 1C there are no growth cones in the caudal SC, and a greater density of growth cones at the rostral end, thus leading to a larger density compensation factor there (Fig. 1E). This density compensation factor is then added to the branching inhibition factor (Fig. 1D) to give the total branching inhibition (Fig. 1F). It can be seen that the minima of the total branching inhibition for the temporal RGC (1) and the nasal RGC (2) are approximately topographically appropriate.

Thus the density compensation factor depends on the locations of the terminals, and the locations of the terminals depend on the density compensation factor. To understand the effect of this feedback loop, a discrete simulation, based on Gierer's, is used (Fig. 1G). At the start of the simulation, 240 RGCs, each with 16 terminals, are allocated to 240 SC cells randomly. (For clarity, only two RGCs, each with four terminals, and eight SC cells are displayed in Fig. 1G.) At each time step, a terminal is chosen at random (red filled synapse of nasal RGC). If the total branching inhibition (indicated by red filled bars) in either neighbouring SC cell is lower, the terminal moves to the neighbour with the lowest branching inhibition (indicated by arrow). The numbers of terminals in each SC location are updated and the compensation factor at each location is then increased in proportion to the number of terminals there. In simulations described later, the compensation factor decays over time, leading to a weaker form of compensation.

**Figure 2:**
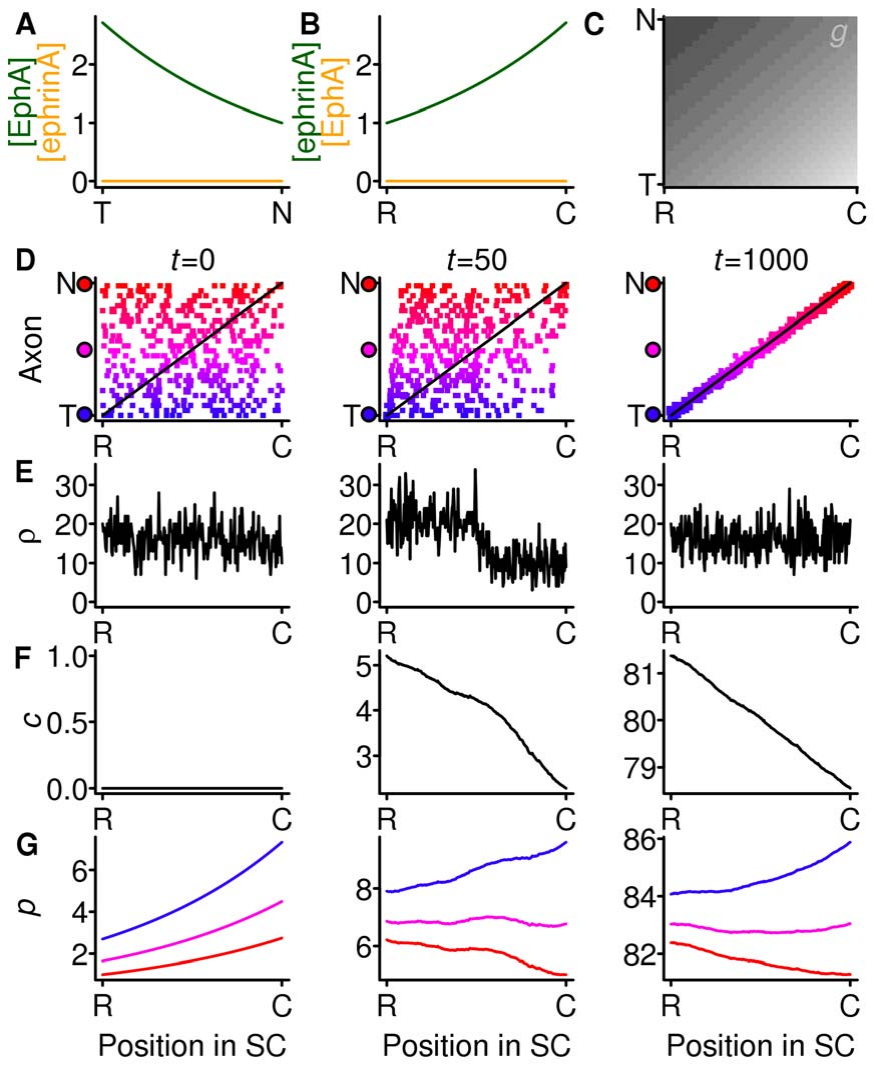
Gradients with strong compensation and no countergradients. **A, B** The gradients (green) of EphA in the retina and ephrin-A in the SC and the countergradients (orange, set to zero) of retinal ephrin-A and EphA in the SC. **C** The branching inhibition throughout the SC for axons from all locations along the nasotemporal axis of the retina. Lighter shading indicates more inhibition. **D**–**G** The time evolution of the mapping. Each column indicates the mapping at one instant. **D** The locations of terminals from the retina (*y*-axis) to the SC (*x*-axis). The retinal origin of the axons is indicated by the continuous shading from nasal (red) to temporal (blue). **E** The number of terminals 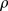 connected to each SC cell. **F** The level of the branching inhibition 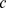 due to density compensation throughout the SC. **G** The value of the total branching inhibition 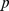 for three axons, whose retinal origin is indicated by the colour of the filled circles on the *y*-axis of **D**. Gradient parameters (see Table 1 for explanation): 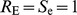, 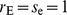. Countergradient parameters: 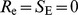. Decay parameter 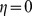 and 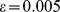.

Given the widespread view that the Gierer model requires gradients and countergradients, it is worth considering, as Gierer did [24], the effect of strong compensation with gradients but no countergradients (Fig. 2). Because only the forward signalling gradient system is present (Fig. 2A,B), all axons are less inhibited at the rostral end of the SC, and throughout the SC an axon is more inhibited the more temporal its origin (Fig. 2C). In Fig. 2D the mapping from a sample of the 240 axons onto the SC is shown at three points in time. Initially (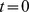) there is a random mapping from axons to the SC. The density at each SC location (corresponding panel in Fig. 2E) has a mean value of 16 (the number of terminals per SC cell), but there are fluctuations, meaning that some locations receive more branches than others. The density compensation factor is set initially to zero throughout the SC (Fig. 2F). The branching inhibition for each axon is shown in Fig. 2G, with the colours of the three curves indicating a temporal axon (blue), a nasal axon (red) and an axon midway along the NT axis (purple).

Later on (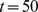) the mapping and the density curve (Fig. 2D,E) show that terminals are more densely packed in the rostral half of the SC. This is reflected in the density compensation factor (Fig. 2F), which is also beginning to build up at the rostral end, causing small shifts in the locations of the minima of the total branching inhibition curves (Fig. 2G). By 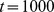 an ordered mapping has emerged, and the density of terminals in the SC is uniform, with fluctuations. This is because the density compensation curve has become more pronounced and shifted the locations of the minima of the total branching inhibition curves to their correct locations.

In summary, it can be seen that a mapping does develop, despite the fact that initially all axons are attracted towards the rostral edge of the SC. This happens because the slope of the branching inhibition experienced by nasal axons (red axons in Fig. 2), which bear the least EphA, is smaller throughout the SC than the slope of the branching inhibition of temporal axons (in blue), which bear more EphA. The increase in the compensation factor that occurs at the overpopulated end of the SC is therefore relatively more important to the nasal axons than to the temporal ones, and is sufficient to displace the minima of their branching inhibition curves to the caudal SC. In the final, ordered, mapping, the temporal (blue) axons experience more repulsion throughout the SC than do the nasal (red) ones, but nevertheless the minima are arranged in an ordered fashion.

The complete absence of the reverse signalling molecules shown in Fig. 2A,B has not been obtained experimentally - it would require conditional knock-out of all the ephrin-A subtypes from the retina and all the EphA subtypes from the SC. However, the same simulation results are obtained if the reverse system molecules are abolished in either the retina or the colliculus since the reverse component will be removed from the branching inhibition (Equation 1 in the Models section). By symmetry, perfect maps would also result from knocking out either the retinal or SC forward signalling molecules. There is no mutant in which either the forward or reverse pathway has been eliminated completely.

The mutant whose gradients resemble removal of countergradients in one structure most closely is the unconditional ephrin-A5 knock-out [14]. As ephrin-A5 is the only graded ephrin-A present in the eye, there is no countergradient of ephrin-A5 in the eye, although there is a constant level of the residual ephrin-A3 and ephrin-A2. There is still a gradient of ephrin-A2 in the colliculus, albeit a weak one with a peak towards the caudal end. In the EphA7 knock-out, the countergradient of EphA in the SC is weakened though not entirely removed [12]. In both these mutants there are mapping errors with ectopic termination zones. This suggests that the model's mapping is better than expected and that this might be due to the effect of the strong form of compensation.

### Weak Compensation with Gradients but no Countergradients Produces a Distorted Mapping

In Gierer's model [24] the concentration of the compensatory substance can only ever increase. This idealised form of compensation has an infinitely long memory of the density of connections in the target region, making it strong, but also biologically implausible. I therefore modified Gierer's model so that the compensatory substance decays in proportion to its concentration (see Equation 2 in the Models section), giving a weaker form of compensation.

**Figure 3:**
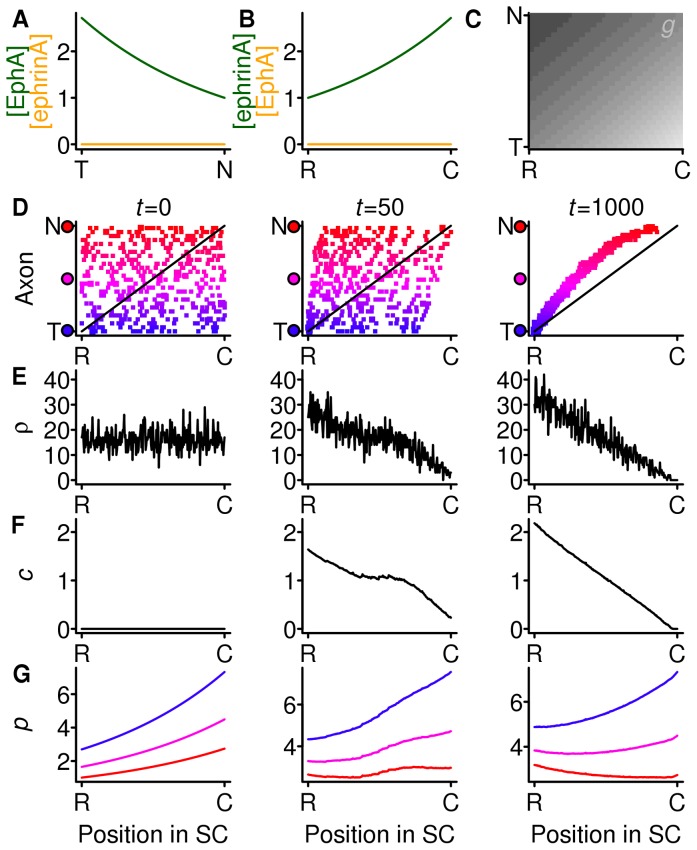
Weak compensation with gradients but no countergradients. The gradients and countergradients are the same as in Fig. 2 but there is now weak instead of strong compensation. Meaning of panels as in Fig. 2. In the final mapping (**D**, 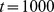) terminals are displaced rostrally from the ideal mapping, indicated by the solid line. Gradient parameters: 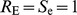, 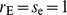. Countergradient parameters: 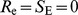. Decay parameter 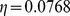 and 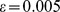.

Fig. 3 shows the effect of replacing the strong compensation employed in the previous simulation with gradients and no countergradients (Fig. 2) with this weaker form of compensation. The mapping starts to develop (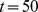) in a similar fashion as when there is strong compensation. However, in the final mapping (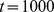) terminals are shifted rostrally from their ideal positions, and the density of connections at the caudal end of the SC is much lower than at the rostral end (Fig. 3E). The compensation factor (Fig. 3F) has reached a steady state, and its range is slightly less than when there is strong compensation (Fig. 2), meaning it has less power to spread out the terminals appropriately. This demonstrates that if the compensation is weak, it is not able to overcome the rostral bias conferred by having only gradients and not countergradients.

The shifted projections are reminiscent of the shifted projections observed in unconditional ephrin-A knock-outs [14], [19], albeit in the opposite direction. Also, in the model there is only one termination zone from each retinal location, in contrast with the experimental ephrin-A knock outs, in which multiple termination zones are found in the SC from some retinal DiI injections. Had the countergradient system (retinal ephrin-As and SC EphAs) been present and the gradient system (retinal EphAs and SC ephrin-As) been knocked out, the situation would have been similar except for the shifts in the terminals being in the caudal direction.

### Addition of Weak Countergradients to Gradients with Weak Compensation Improves the Mapping

**Figure 4:**
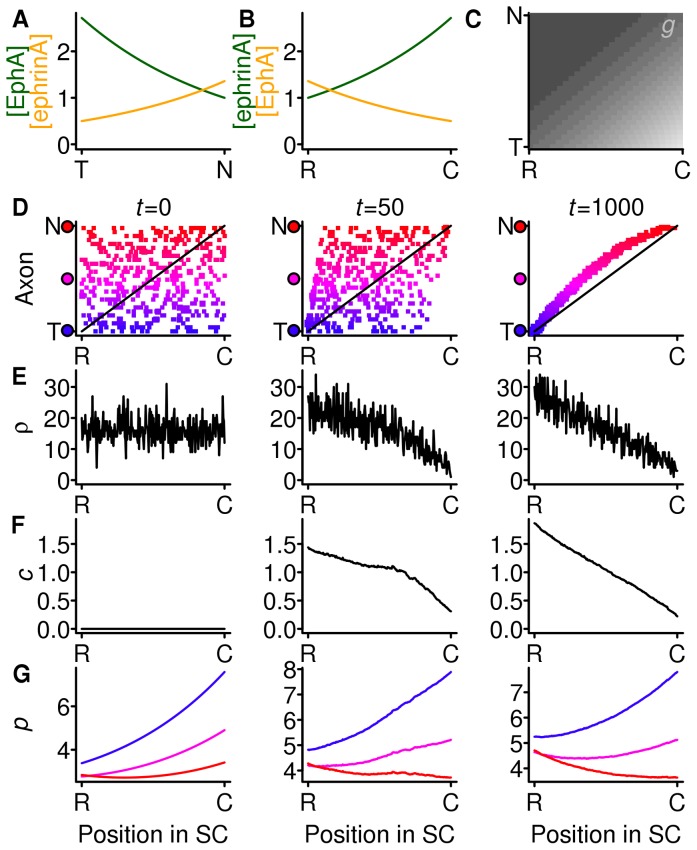
Addition of weak countergradients to gradients with weak compensation. The gradients and level of compensation is the same as in Fig. 3 but weak countergradients (orange) have been added. Meaning of panels as in Fig. 2. In the final mapping (**D**, 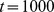) the rostral displacements from the ideal mapping are smaller than in Fig. 3. Gradient parameters: 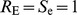, 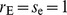. Countergradient parameters: 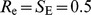, 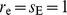. Decay parameter 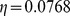 and 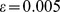.

In the presence of strong compensation, the addition of countergradients does nothing to improve the mapping (simulations not shown), since gradients and strong compensation already give rise to a perfect mapping (Fig. 2). However, with weak compensation and gradients without countergradients (Fig. 3) the mapping is shifted. To investigate if countergradients could have a function when there is weak compensation, I added weak countergradients, half the height of the gradients (Fig. 4A,B), a combination that without compensation would be expected to produce a shifted mapping. In the final mapping (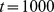) there are still rostral shifts, though less pronounced than without any countergradients (Fig. 3). The countergradient has acted in concert with the compensation mechanism to produce a mapping that is more towards the ideal map. The combination of gradients, countergradients and compensation in this simulation gives the closest approximation to the wild-type phenotype of the simulations presented so far: the mapping is reasonable (Fig. 4), and it is distorted by knocking-out the countergradients (Fig. 3).

### Gradients, Countergradients and Weak Compensation can Produce ki-ki and Math5 Phenotypes

A good model of retinotopy should be able to reproduce, at least qualitatively, the phenotypes produced by experimental genetic manipulations when analogous manipulations are applied to the model. I therefore tested whether a model with gradients, weak countergradients and weak compensation can reproduce the EphA3 knock-in [15]–[17] and Math5 knock-out [32] phenotypes.

**Figure 5:**
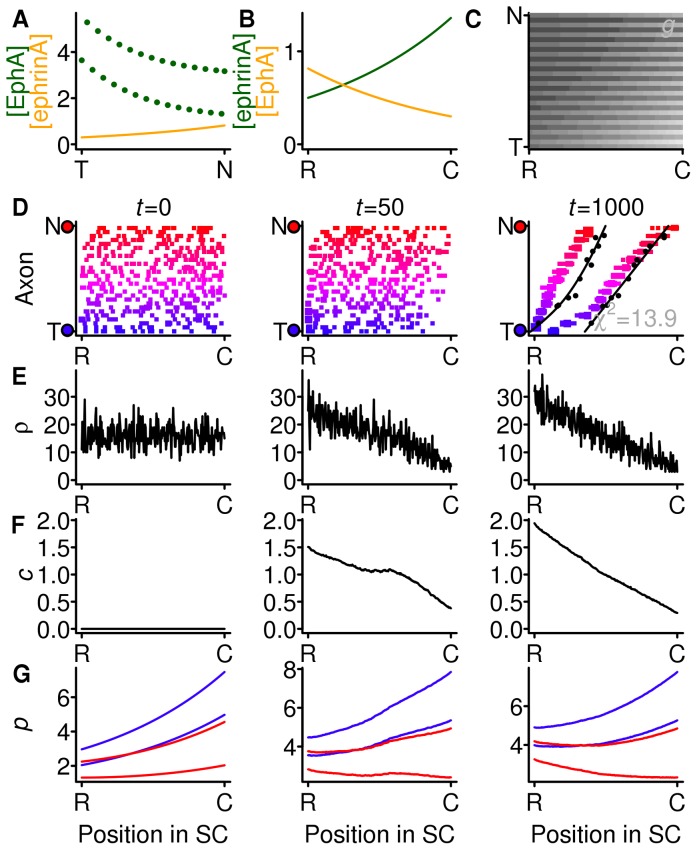
EphA homozygous knock-in simulations. Meaning of panels as in Fig. 2. Experimentally measured retinal EphA gradients (**A**, green dotted lines) are used. The upper gradient is found in 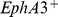 RGCs, in which EphA3 has been knocked in and the lower gradient is from the 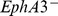 RGCs which have no EphA3 (see Table 2, 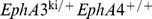 for parameters). In the simulations the extra EphA knocked into alternating RGCs leads to the RGCs bearing more EphA experiencing greater branching inhibition (**G**) and there being two maps (**D**, 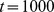), the rostral-most map being from the 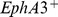 RGCs. The experimental maps are indicated by the black points in **D**, 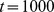 and a nonparametric regression fit to the data is shown with a solid line; for clarity, the standard error in the mean of the fit is not shown. The goodness-of-fit 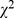 between the experimental and simulated data is also indicated in grey (see Models section for explanation). SC gradient parameters are as in previous figures: 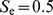, 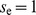. Countergradient parameters: 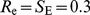, 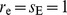. Decay parameter 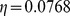 and 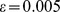.

In EphA3 knock-in mice, a constant amount of EphA is knocked into around 40% of RGCs randomly throughout the retina [15]–[17], leading to a phenotype in which there are double maps, one each from the wild-type and knocked-in RGC populations. I simulated this by ''knocking-in'' some EphA in to every second axon, as shown in Fig. 5. The parameters of the retinal EphA gradients and the amount of EphA3 knocked in were taken from in-situ hybridisation experiments [16]. The extra EphA3 gives two sets of EphA gradients in the retina, and a double map reminiscent of that found experimentally [15], [16] develops. Double maps also form when strong compensation is present (simulations not shown). These results indicate that weak compensation can confer the kind of flexibility needed to redirect terminals to positions to which they would not project normally.

**Figure 6:**
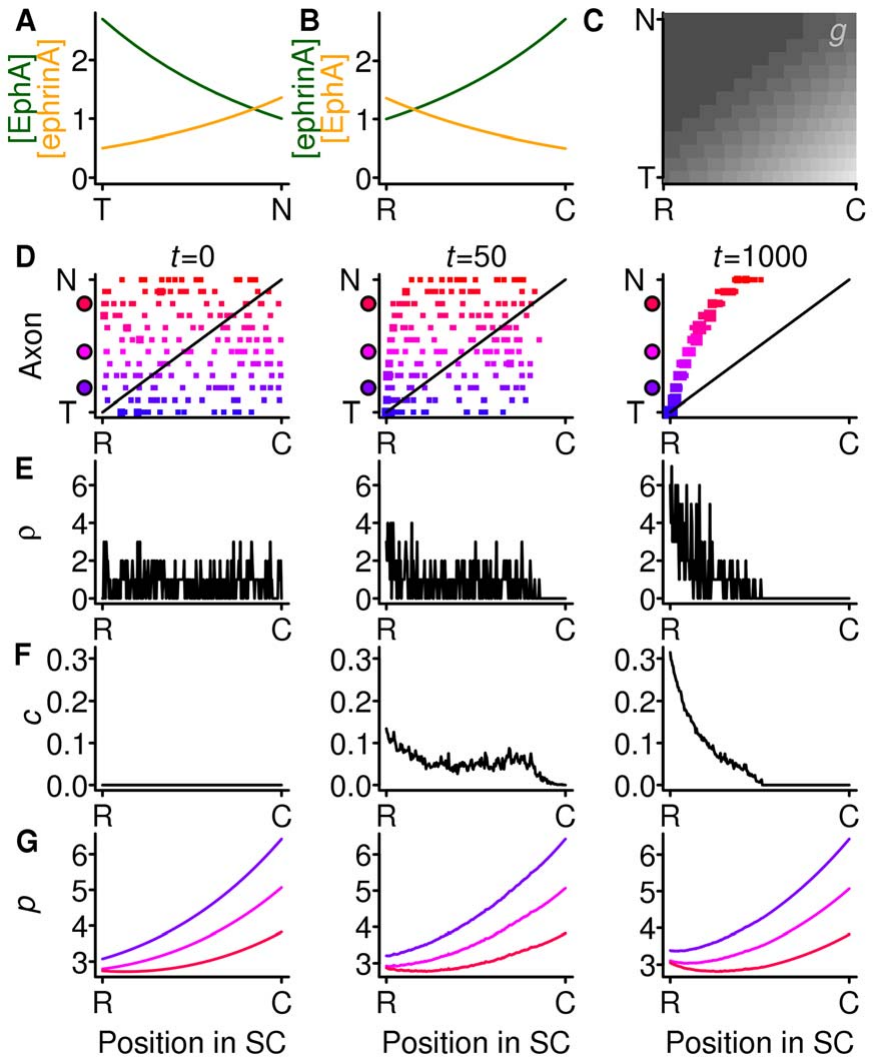
Math5 knock-out simulations. Meaning of panels as in Fig. 2. There are 5% of the number of RGCs in all the preceding simulations and all other parameters are identical to Fig. 4. A mapping that does not cover the entire SC develops (**D**, 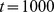), in contrast to the mapping shown in Fig. 4D.

The Math5 knock-out [32] has approximately 5% of the number of RGCs of a wild type, roughly evenly distributed across the retina. This leads to the density of termination zones being higher in rostral SC than caudal SC. I examined whether a model with gradients, weak countergradients and weak compensation could reproduce this behaviour by removing 95% of the RGCs in the model. The resulting ''phenotype'', with the same set of mismatched gradients used in Fig. 4, is shown in Fig. 6. The map covers the rostral third of the SC (Fig. 6G, 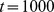), a coverage that is actually considerably lower than the biological phenotype. However, this does demonstrate how the model works: with fewer axons there is less pressure on terminals to move away from the favoured locations at the rostral end of the SC.

**Figure 7:**
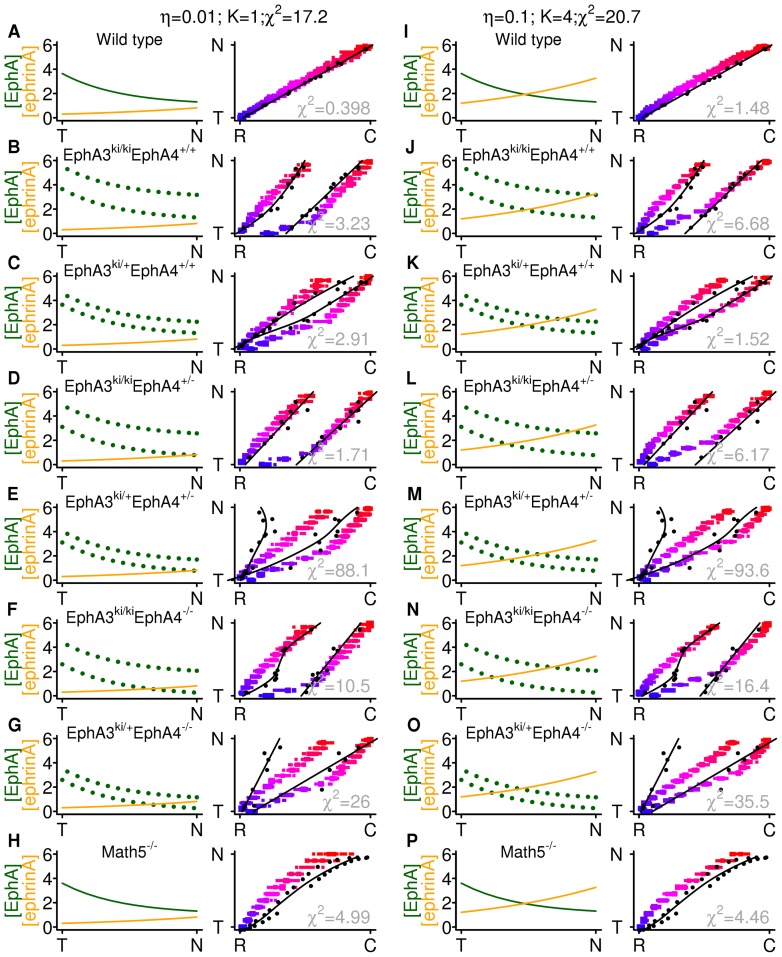
Two sets of mutant simulations. **A** Wild type gradients (left) and location of RGC terminals in SC (right) at end point (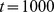) of simulation with decay parameter 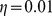 and same level of countergradients as in Fig. 5. The experimental map is indicated by original data (points) and nonparametric regression fit (solid line); for clarity, the standard error in the mean of the fit is not shown. The goodness-of-fit 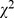 between the experimental and simulated data is also indicated in grey (see Models section for explanation). **B**–**G** The six combinations of EphA3 knock-in and EphA4 knock-out in same format as (A). **H** The Math5 knock-out, with 5% of the number of RGCs as the wild type (A). **I**–**P**. Corresponding simulations to A–H, but with retinal countergradients four times as strong and weaker compensation (due to the tenfold larger decay parameter 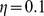). Parameter 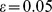 in all simulations.

As well as the homozygous knock-in of EphA3 (as modelled in Fig. 5), there are heterozygous knock-in mice, in which half as much EphA3 is knocked-in [15]. These knock-in mice have been bred with heterozygous and homozygous EphA4 knock-out mice [16], in which EphA4, normally expressed uniformly along the nasotemporal axis of the retina, is either absent or expressed at half its usual strength. There are thus six combinations of combined knock-in and knock-out mutants, each of which has had the map along the nasotemporal axis measured anatomically [15], [16]. Along with the wild type and the Math5 knock-out, this gives a set of 8 maps against which to test the model. In each map apart from the Math5 knock-out, the retinal EphA gradients and the amount of EphA3 knocked in have been measured using in-situ hybridisation experiments [16]; I assume that gradients in the Math5 knock-out are the same as in wild types. Fig. 7A–H shows the gradients (equations for which are in Table 2) and resulting maps for each mutant. The countergradients (not shown) are the same as in Fig. 5 and the compensation is stronger. The fit for most maps is good, as indicated by the goodness-of-fit measure 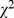 (see Models section for definition). The two mutants with obviously bad fits are the heterozygous knock-ins with heterozygous or homozygous knock-out of EphA4 (Fig. 7E,G). Here the axons with extra EphA3 are not shifted as rostrally as they are in the experiments.

**Table 2:** Retinal EphA gradients for knock-in simulations.

Genotype	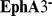	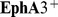
Wild type	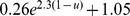	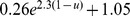
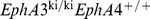	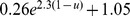	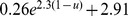
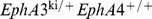	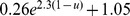	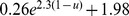
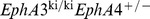	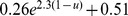	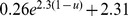
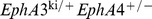	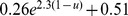	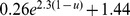
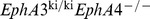	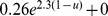	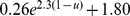
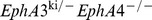	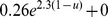	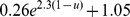

The EphA retinal gradients used in the various genotypes modelled in Figs. 5 and 7 in RGCs that do not have EphA3 knocked in (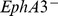) or that do have EphA3 knocked in (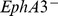).

In three of the mutants (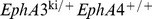, 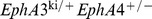, and 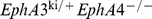) experimental DiI injections show that there are two maps, though towards the rostral end of the SC the two maps appear to merge, or ''collapse'' [16]. The corresponding simulations (Fig. 7C,E,G) show that the distributions of terminals from neighbouring temporal 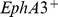 and 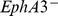 RGCs do overlap along the rostrocaudal axis; in a simulated DiI injection experiment, in which the terminals of a number of RGCs within a radius of the injection site are labelled, this might give the appearance of a single termination zone, as seen in the experiments. However, this is not the strict ''collapse'' which occurs in some models that include the effect of spatially correlated activity in the retina and an implicit synaptic plasticity mechanism [29], [36]; in these models the mean locations of branches from neighbouring 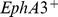 and 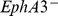 RGCs are indistinguishable at the temporal end of the map. This strict collapse will not occur in the same way in the generalised Gierer model due to the lack of activity in the model.

The simulations earlier in the paper have suggested a hypothesis that how well gradients and countergradients are matched can be traded off against the strength of compensation in order to produce good wild type maps. In order to explore whether this holds for a range of mutants, I varied both the strength of compensation and the size of the retinal ephrin-A countergradients. For each combination I recorded a total goodness-of-fit parameter and plotted it in the 2D parameter space formed by the compensation strength and the countergradient size (not shown). I found that for any particular size of countergradients, there was an optimal strength of compensation; the larger the countergradients, the weaker the compensation required for the best set of maps. Fig. 7I–P shows the simulation results for countergradients 4 times as strong as those in Fig. 7A–H and with compensation 10 times as weak, as measured by the decay factor. The overall fit is 17% worse (see Models for definition of goodness-of-fit measure), but for each phenotype the fit is still reasonably good apart from in the heterozygous EphA3 knock-ins with heterozygous or homozygous knock-out of EphA4 (Fig. 7M,O).

## Discussion

### What is the Role of Countergradients?

Gierer's 1983 model [24] was devised to account for the compression [38] and expansion [39], [40] of maps in goldfish. I have applied the model to investigate the relative functional importance of countergradients and compensation mechanisms in mice. Provided there is a sufficiently powerful compensation mechanism, countergradients are not needed for an ideal retinocollicular map to develop. This is contrary to the experimental results obtained when part of the countergradient system (EphAs in the SC) is knocked out [12] : mapping deficits occur. When both gradients and countergradients are reduced by increasing the amounts of ephrin-As knocked out, mapping deficits also occur and get more severe [14], [19], [21]. Thus the perfect mapping obtained in the model with no countergradients and strong compensation suggests that there is not strong compensation in the biological system. However, when a more realistic form of weak compensation is present, addition of countergradients does improve the mapping, suggesting that countergradients, along with a limited form of compensation or other adaptive mechanism, are required for the wild type map to develop.

I have shown that when the model with weak compensation is given the gradients which are present in homozygous EphA3 knock-in genotypes [15], [16] along with weak countergradients, it is able to reproduce a double map that resembles the phenotype. Double maps can also result from simulations with no countergradients. However, even if the base gradients and countergradients are matched, some degree of compensation is required to remap the axons with extra EphA knocked in. In a simulated Math5 knock-out, in which a large fraction of RGCs are absent, with unmatched gradients and countergradients the retinal map can cover a fraction of the SC, rather than whole SC when the full complement of RGCs are present. This is actually a more extreme phenotype than is observed along the rostrocaudal axis experimentally [32], but demonstrates the effect of fewer axon terminals releasing less inhibitory substance, allowing axons to settle towards the end of the SC favoured by chemospecific cues.

The wild type simulations in which compensation and countergradients were varied lead to the hypothesis that the ratio of countergradient to gradient strength and the strength of compensation can be traded off against each other: the stronger the countergradients are relative to gradients, the weaker the compensation needs to be to obtain wild type maps, and vice-versa. To test this more rigorously, I ran the model with various levels of countergradients and compensation on a set of retinal EphA gradients for wild type, Math5 knock-out and combinations of hetero- and homozygous EphA3 knock-in and EphA4 knock-out, measuring the goodness-of-fit between the simulated maps and maps measured in experiments [15], [16]. The fits were not good for all phenotypes, but the hypothesis was confirmed: stronger countergradients meant that weaker compensation was needed for the best set of maps.

Within the Gierer framework the conclusion that gradients, weak countergradients and weak compensation is sufficient to explain a range of data from mouse does not necessarily extend to other species. In goldfish the terminals of regenerating nerves from a temporal hemiretina first occupy rostral tectum and then, over a period of months, expand to fit the tectum [39]. Conversely, regenerating nasal axons occupy caudal tectum initially [1] and gradual spreading of these fibres is also reported [41]. These results imply that the gradients and countergradients in fish are matched. However, this reasoning is within the framework of fixed gradients, and theories involving respecification of the gradients [30], [39], [42], [43] would not require matching of gradients. Likewise, in zebrafish the results from experiments in which the effect of competition has been removed [44] suggest that fairly well-matched gradients and countergradients would be needed. However, this experiment was undertaken at one developmental stage; and in zebrafish the area of the tectum increases over 100-fold whilst innervated by retinal axons [45], raising the question of how stable the gradients actually are.

### Is Gierer's Compensation Mechanism Supported by Data?

The assumption behind Gierer's strong compensation mechanism - that molecular mechanisms of synapse formation and destruction will tend to maintain equal numbers of synapses onto target cells - is reasonable. However, strong compensation can be ruled out, since the build-up of inhibitory substance in proportion to the density of terminals over time without any decay is biologically implausible. It also leads to a perfect map in the case of gradients without countergradients (Fig. 2). A weaker and biologically plausible form of compensation, with decay over time, produces distorted maps in wild types with mismatched gradients and countergradients, but cannot product the ectopic projections observed experimentally in ephrin-A and EphA knock-outs.

The molecular identity of neither density compensation nor competition is known, though the BDNF-TrkB pathway has been put forward as a candidate to implement competition [16]. Alternatively, if a SC neuron releases BDNF when it has fewer than a target number of inputs, this could be viewed as a form of density compensation, albeit with an attractive rather than inhibitory cue. Another mechanism that may have a similar effect to density compensation is homoeostatic plasticity [46], whereby the total synaptic strength onto a postsynaptic neuron is regulated.

### Do the Model Simplifications and Data Limitations Matter?

Do any of the simplifications inherent in the model and limitations of the experimental data invalidate the conclusions drawn above? A potentially critical simplification is the reduction of the geometry of the retina and the SC from two-dimensional manifolds to one-dimensional lines. The justification for this is twofold. (1) The EphA/ephrin-A family are aligned approximately with the nasotemporal and rostrocaudal axes of the retina and the SC respectively, whereas the EphB/ephrin-B family are aligned approximately with the dorsoventral and mediolateral axes. (2) Simulations have been carried out which demonstrate that the powerful spreading action of the compensation mechanism also occurs in 2D, including a case when there are gradients and no countergradients along each axis [47].

A second important simplification is that activity and activity-dependent plasticity are not considered in the model. Clearly activity plays a role in the development of the mapping from the retina to the SC, though it is thought to be more important for refining projections that have been structured roughly by other mechanisms [27]. The mapping obtained in the model without countergradients and with weak compensation (Fig. 4) is still more ordered than the experimental mappings, in that there are none of the ectopic projections present in most knock-out phenotypes [12], [14], [19]. It is possible that the addition of an activity mechanism might lead to the production of ectopics [48].

Modelling of the gradients is limited by the data available. I have made educated guesses about the profile of retinal ephrin-A and SC EphA and ephrin-A gradients since they have not been measured quantitatively, as have the retinal EphA gradients [16]. This does not affect the qualitative conclusion that countergradients and compensation can coexist and complement each other. However, it may affect the goodness-of-fit found in Fig. 7, since changes in the steepness of the countergradients will lead to a compression or expansion of the branching inhibition profile (see Equation 5 in Models section). Furthermore, it is probable that the various members of the EphA and ephrin-A families bind to each other with different affinities, but lack of reliable quantitative information on the expression profiles means that it is not worthwhile modelling all the EphAs and ephrin-As separately.

### Comparison with Other Models

There are two main classes of chemoaffinity models of retinocollicular mapping [49]. In Type I models [23], [25], [26], [33], [36] each retinal cell has a high affinity for a small group of collicular cells and less affinity for all others. In Type II models all cells have the highest affinity for one end of the SC. To produce a map, Type II models require some additional mechanism such as competition [32], [49], activity [29], [48], [50] or marker induction [30], [43]. The marker induction model differs from all the other models in that the gradients in the target region are not fixed, flexibility being achieved by ingrowing fibres inducing these gradients.

The model presented here can be set up either as a Type I model, with matched gradients and countergradients, or as a Type II model, with gradients and no countergradients. The intermediate case, with mismatched gradients and countergradients, is a Type I model in the sense that each retinal cell has a collicular cell of maximum affinity, but the collicular cell with which it has maximum affinity is not the topographically ''correct'' cell. Although the model presented here has fixed gradients, it could be that the density compensation is implemented by modification of EphA and ephrin-A gradients, in which case it would be a form of marker induction [30].

A recent proposal [34] does not fit the strict definition of either Type I or Type II models. Here each retinal cell has an almost equal affinity for a relatively large group of collicular cells (of the order of 50% of one axis of the SC) and virtually no affinity for other cells. Initially, branches are formed in the regions permitted by these affinities, and then an activity-dependent process refines the connections. There are two problems with this proposal. First, there is no mechanism to relate the relatively gentle gradients of Ephs and ephrins into the box-shaped affinity functions proposed. These affinity functions have to be constructed independently of gradients, meaning that there is no principle apparent in the affinity functions used to model the EphA3 knock-ins. Second, the model does require an affinity function that gives a rough wild-type map to work; in this sense the model is closer to the Type I models.

The recent model of Triplett et al. [32] has a number of similarities with the generalised Gierer model. In the Gierer model the number of terminals per axons is fixed, whereas in the Triplett et al. model, it is encouraged to lie around a particular value. In the Triplett et al. model a ''competition'' term penalises each SC cell in proportion to the square of the number of terminals on it, whereas in the generalised Gierer formulation, the penalty (due to the inhibitory substance) is directly proportional to a moving average of the number of terminals on the SC cell. In the limit of a very small averaging time window, the models will be identical apart from the non-linearity in the Triplett et al. model and how the adaptive mechanisms and fixed molecular mechanisms are scaled relative to each other.

A recent model [35] appears have the flexibility inherent in Type II models with some sort of compensatory mechanism. Here the interactions between Ephs and ephrins on the ingrowing retinal axons (fibre-fibre interactions) can help to spread out the mapping in the face of mismatched gradients. However, it appears that for this to happen, the relative strengths of the forward and reverse signalling pathways have to be tightly controlled.

### Challenges in Understanding Mechanisms of Retinotopy

While much experimental data has been collected in recent years, existing models have not been tested rigorously against the data [37]. A necessary condition to have explained the development of retinotopy in mouse is to have created a model that, with suitable changes to gradient parameters, can account for the knock-in phenotypes and the many knock-out phenotypes in the literature [7], [12]–[16], [18]–[22]. This requires an adequate characterisation of the experimental data and a measure of goodness of fit between a model and an experimental phenotype. This is particularly challenging for the knock-out phenotypes because the anatomical mapping data is not comprehensive in the sense that in any one animal only a few injections of tracers are possible, so the data obtained in any one animal is only a very small section of the mapping. The variability of the arborisations of RGCs from the same retinal locations between ephrin-A knock-out individuals will probably confound attempts to construct a composite map across individuals, as has been done in the knock-in phenotypes [15], [16]. Functional mapping techniques [21] overcome the problem of obtaining an entire map, but methods are needed to detect and quantify ectopic termination zones using these methods.

A successful model will be sensitive - but not too sensitive - to the relative strengths of multiple mechanisms. At present, no one model with the same set of parameters satisfies this condition. I hope this paper has illustrated the challenges implicit in finding such a model.

## Models

The mathematical details of the model, a generalised version of Gierer's (1983) model, are presented here; for justifications of its elements see the Results and Discussion.

### Gradients and Countergradients

There is a concentration [EphA]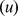 of EphA and a concentration [ephrinA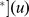 of ephrin-A at point 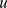 along the nasotemporal axis of the retina; 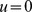 is the temporal pole and 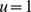 is the nasal pole (Fig. 1A). Along the rostrocaudal axis of the SC there are concentrations [ephrinA]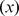 and [EphA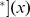 of ephrin-A and EphA respectively; 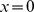 is the rostral pole and 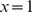 is the caudal pole (Fig. 1B). The shapes of gradients are described by exponential curves and the height and slope of each curve is a free parameter, making eight parameters in total (Table 1).

### Branching Inhibition due to Molecular Gradient Signalling

For a terminal belonging to an axon originating at a point 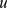 along the nasotemporal axis, the branching inhibition 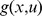 due to forward and reverse EphA to ephrin-A binding it experiences at point 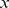 along the rostrocaudal axis of the SC (Fig. 1D) is:
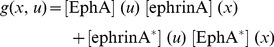
(1)


There are other expressions that could be used for 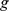, for example ones involving receptor or ligand saturation [23], [29].

### Branching Inhibition Due to Density Compensation

The branching inhibition due to density compensation 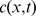 experienced by all terminals at a time 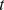 at a point 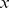 in the SC (Fig. 1E) depends on the density 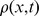 of connections in that region of the SC:
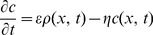
(2)where 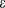 specifies how quickly 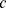 changes in response to the density and 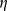 parametrises the rate of decay. In simulations with strong compensation 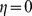. When there is weak compensation 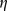 is a positive number and a steady state could arise, in which 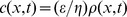.

### Total Branching Inhibition

The contributions to branching inhibition from molecular gradient signalling and density compensation are summed to give the total branching inhibition 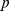:
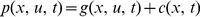
(3)


The dynamics of the system, as described below, mean that an axon originating from location 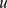 will tend to move towards a location 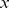 that minimises its total branching inhibition 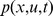.

### Discrete Implementation

So far, for ease of notation and algebraic manipulations to be presented later, the model has been formulated as though the SC were a continuous medium. It is of course a collection of discrete neurons and the mapping is from individual RGCs 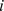 to SC cells 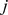. The continuous representation can be translated into a discrete one by denoting the positions of RGC 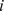 as 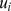 and the position of SC cell 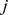 as 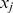; the quantities 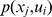 and 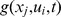 can be abbreviated 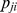 and 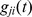.

At the start of the simulation the 16 terminals of each RGC are allocated to SC cells randomly. At each time step, a terminal is chosen at random; suppose that the terminal is on SC cell 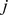. If the branching inhibition in either neighbouring SC cell is lower, the terminal moves to the neighbour with the lowest branching inhibition. After moving, the values of the density 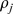 and 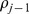 or 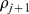 are updated. The compensation factor 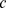 is then updated for all locations 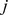:
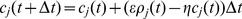
(4)where 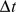 is set to the reciprocal of the total number of terminals (i.e. the product of the number of axons and the number of terminals per axon). This scaling should mean that the mapping progresses at the same apparent rate in systems of differing sizes. The update scheme that Gierer [24] used is not clear from his paper, but the endpoints of the results I obtain are the same as his; a more detailed discussion is available elsewhere [51]. All simulations and analysis were carried out in R [52] and the code is available in the supporting information (Dataset S1).

### Matching Gradients

The end result of a successful mapping mechanism should be a map in which axons along the nasotemporal axis of the retina are mapped onto the rostrocaudal axis of the SC. With our definition of 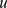 and 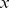, this means that the destination of an axon originating from 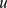 should be 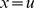. For any particular form of gradients of retinal and SC Ephs and ephrins, we can compute the expected mapping from the retina to the SC. The assumption of exponential gradients [23] allows for simple mapping formulae in terms of eight parameters, the heights (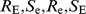) and decay or rise constants (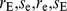) of each of the four exponentials (Table 1). Given that each axon tries to find the position of minimum branching inhibition, the optimal position of an axon originating from location 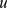 can be computed by substituting the expressions for the EphA and ephrin-A concentrations into Equation 1 and finding the value of 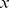 for which the derivative of 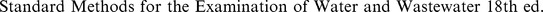 with respect to 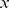 is zero. This calculation yields:
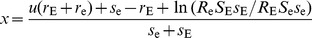
(5)


From this formula it can be seen that a perfect mapping 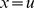 can be formed by setting all the heights to the same value and all the decay and rise constants to the same value. There are also an infinite number of parameter settings in which the mapping is shifted and expanded or contracted.

### Goodness-of-fit to Experimental Data

The wild type and EphA3 knock-in experimental data sets comprise pairs 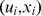 of nasotemporal sites and the location of the corresponding termination zone in the SC. The Math5 knock-out data set is strictly a profile of the cumulative intensity of dye in the SC following a whole-eye injection, and I have interpreted this as forming a map. Nonparametric regression with a local-linear estimator and Kullback-Leiber cross-validation as implemented in the R np package [53] was used to estimate for distance along the nasotemporal axis 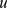 the distance of the injection along the rostrocaudal axis 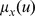. The nonparametric regression also gave an estimate of the error in the mean 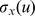. From the simulation results, the weighted mean location 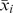 of the terminals from RGC 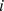 was found using the formula 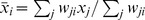 where 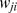 is the number of terminals from axon 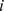 on SC cell 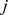. The goodness-of-fit between a theoretical map and an experimental map was defined:
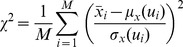
(6)where 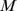 is the number of RGCs. For mutants in which there were double maps, 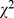 was computed separately for the 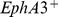 and 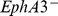 maps, and the resulting 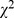 values were averaged. For the entire set of mutants (Fig. 7) the 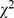 values were averaged to give an overall value.

## Supporting Information

Dataset S1 **Complete source code for the simulations.** This allows the simulations underlying Figs. 2, 3, 4, 5, 6, 7 in this paper to be run and the results plotted.
(ZIP)
