# Correction: On the Importance of Countergradients for the Development of Retinotopy: Insights from a Generalised Gierer Model

**DOI:** 10.1371/annotation/6d3b4757-2737-4570-9018-28802eb9437a

**Published:** 2014-01-06

**Authors:** David C. Sterratt

In the fifth sentence of the section "Matching Gradients" of Models, the text "Standard Methods for the Examination of Water and Wastewater 18th ed." is incorrect and should instead read "*g*". 

